# Modified C-V Flap for Nipple Reconstruction

**DOI:** 10.7759/cureus.80256

**Published:** 2025-03-08

**Authors:** Ulises Solis Bermudez, Mariana Gabriela Monroy Velázaquez, Karina Lizbeth Lara Sampayo, Sergio Federico Estrada Tijerina, Jesus Eduardo Trujillo Rodríguez, Moisés Alvir Ruiz Lopez, Erik Ponce Graciano, Oscar Ulises Rosales Martinez

**Affiliations:** 1 Medicine, Universidad Vasco de Quiroga, Mexico City, MEX; 2 Medicine and Surgery, Hospital General de Zona No. 32 Dr. Mario Navarro Madrazo, Instituto Mexicano del Seguro Social (IMSS) Órgano de Operación Administrativa Desconcentrada en la Ciudad de México Sur (OOAD CDMX Sur), Mexico City, MEX; 3 Medicine and Surgery, Hospital General de Zona con Medicina Familiar No. 2, Instituto Mexicano del Seguro Social (IMSS), Mexico City, MEX; 4 Medicine and Surgery, Facultad de Medicina y Ciencias Biomédicas, Universidad Autónoma de Chihuahua, Chihuahua, MEX; 5 Medicine and Surgery, Universidad Cristóbal Colón, Mexico City, MEX; 6 Medicine and Surgery, Universidad Autónoma Benito Juárez de Oaxaca, Oaxaca, MEX; 7 Medicine and Surgery, Hospital General del Sur Puebla, Puebla, MEX; 8 Medicine and Surgery, Hospital General Regional No. 72 Licenciado Vicente Santos Guajardo, Instituto Mexicano del Seguro Social (IMSS) Universidad Nacional Autónoma de México, Mexico City, MEX

**Keywords:** breast, c-v flap, nipple reconstruction, plastic and reconstructive surgery, surgical flaps

## Abstract

Nipple reconstruction is a crucial aspect of breast reconstruction, focusing on restoring the aesthetic appearance of the nipple-areola complex (NAC). Techniques such as the C-V flap are commonly used, but they often face challenges in maintaining nipple projection over time, requiring overcorrection. The modified C-V flap, incorporating purse-string sutures, improves projection retention and reduces the need for revisions. A 63-year-old woman with a history of failed reconstructions underwent a modified C-V flap, achieving favorable results. This technique, combined with composite grafts and tattooing, addresses complications such as radiotherapy effects and enhances the overall outcome. The approach offers a reliable and personalized solution, optimizing long-term results in nipple reconstruction.

## Introduction

Nipple reconstruction plays a vital role in breast reconstruction, with the goal of restoring the aesthetic appearance of the nipple-areola complex (NAC). Several techniques are used, each with its own specific considerations and potential complications. Local flaps are the most commonly employed method for nipple reconstruction, including techniques such as the skate flap, star flap, and C-V flap. These local flaps are preferred due to their reliability and lower complication rates compared to grafts. However, a notable drawback is the tendency for significant loss of projection over time, often requiring overcorrection by 25%-50% to achieve the desired long-term outcome [1,2].

Composite grafts, such as those using tissue from the contralateral nipple, can be combined with local flaps to improve projection and symmetry. This technique, known as nipple sharing, is particularly effective when the contralateral nipple is large and the skin is thin. It has demonstrated favorable results in maintaining projection and achieving symmetry with minimal complications [3].

For patients who have undergone radiotherapy, special precautions are necessary due to the higher risk of complications, such as flap necrosis. To address this, techniques using purely dermal local flaps have been developed, offering a safer alternative for nipple reconstruction in irradiated patients [4].

Tattooing is the most common and safe method for areola reconstruction, often preferred over grafts due to its lower complication rate and satisfactory aesthetic outcomes. While grafts can be taken from areas such as the upper inner thigh, they carry a higher risk of complications compared to tattooing [2,5].

Ultimately, the choice of technique is influenced by several factors, including the patient's anatomy, prior treatments (such as radiotherapy), and personal preferences. The primary objective is to achieve a symmetrical, aesthetically pleasing nipple-areola complex (NAC) with minimal complications and satisfactory long-term results [1,2,6].

The modified C-V flap technique for nipple reconstruction is a surgical method aimed at improving the long-term projection of the reconstructed nipple. This approach incorporates a modified C-V flap with purse-string sutures at the base of the nipple. The key objective of this modification is to address the common challenge of maintaining nipple projection over time [7].

Medical literature indicates that the modified C-V flap with purse-string sutures significantly enhances the retention of nipple projection compared to the traditional C-V flap technique. In a study involving 116 patients, those treated with the modified technique exhibited a higher rate of sustained nipple projection at three, six, and 12 months post-surgery and a lower need for revision procedures than those undergoing the conventional approach. This improvement is attributed to the stabilizing effect of the purse-string sutures on the nipple base, which minimizes projection loss over time [7].

## Case presentation

A 63-year-old woman with a history of invasive ductal carcinoma of the left breast treated with mastectomy, chemotherapy, and radiotherapy presented for her third breast reconstruction. Her previous attempts included an implant-based reconstruction complicated by exposure and removal, followed by a latissimus dorsi flap with implant, which developed grade III capsular contracture. Due to the failure of previous NAC reconstruction attempts and the patient's preferences, a modified C-V flap was planned with a 40% overcorrection to compensate for expected involution and achieve symmetry with the contralateral breast. The flap, harvested from the lateral thoracic region to ensure optimal vascular supply, was meticulously shaped to enhance projection and contour. At six weeks, the expected involution of the flap was observed, achieving an aesthetically pleasing and symmetrical result.

Surgical technique: Modified C-V flap for nipple reconstruction

**Figure 1:**
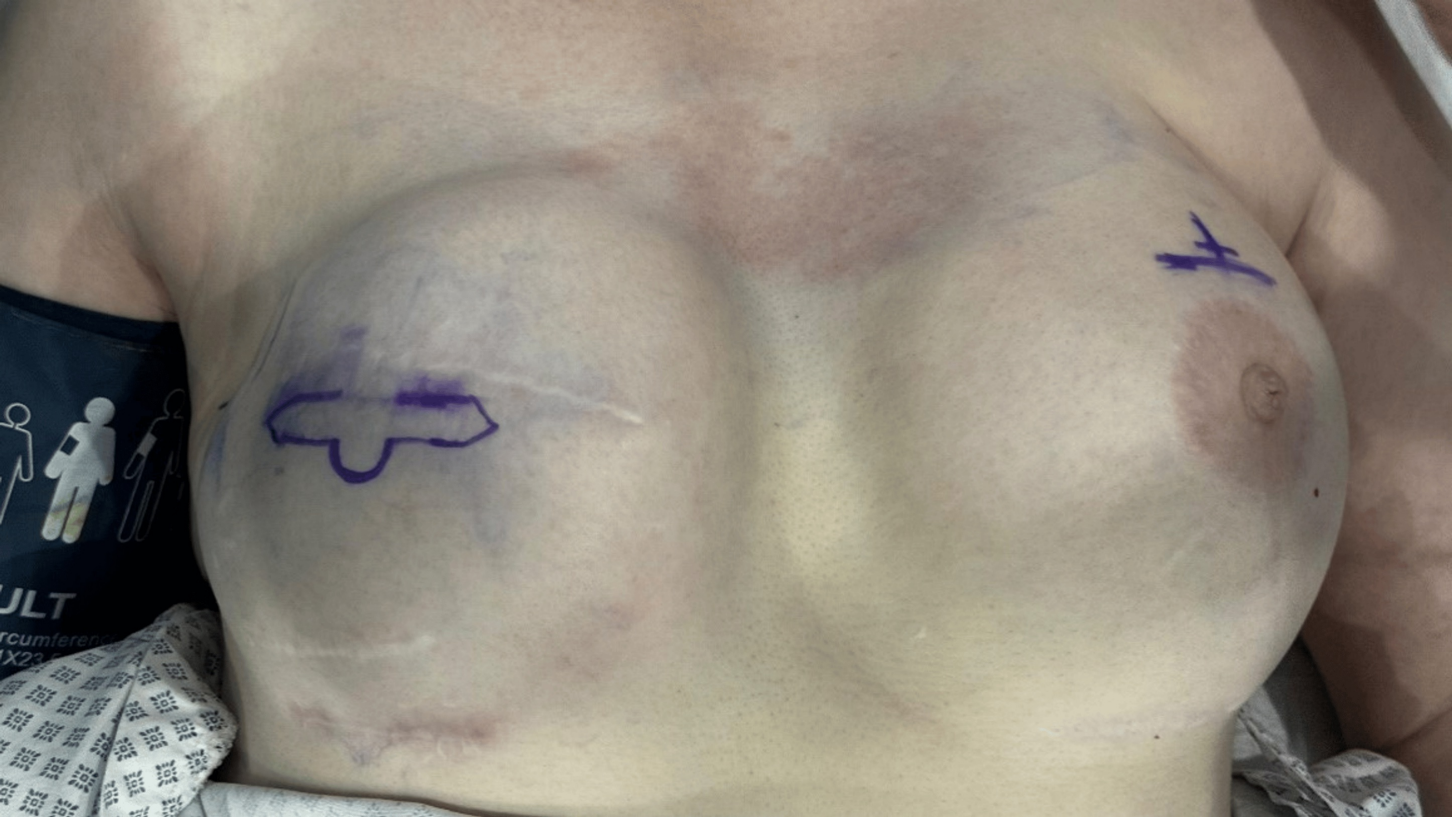
Preoperative flap design

The position of the C-V flap was marked with the patient in a sitting position, ensuring alignment with the location of the contralateral nipple. The flap design considered the previous operative scars, ensuring the unincised skin at the base of the central C flap avoided scarred areas to preserve the blood supply from the subdermal plexus. The design consisted of a central circular C flap and two lateral rectangular V flaps (Figure 1). The diameter of the C flap determined the size of the new nipple, while the projection was defined by the width of the V flaps. To account for postoperative shrinkage, the diameter of the C flap was planned 5%-10% larger, and the projection was designed 20%-30% higher than the contralateral nipple.

After administering local anesthesia, the incisions were made, and the flap was carefully elevated, preserving sufficient subdermal fat. The donor sites of the V flaps were closed subdermally using 3-0 Monosyn sutures. In the modified technique, subdermal purse-string sutures were placed around the margin of the C flap donor site using 3-0 Prolene to reduce the diameter of the nipple base, shaping it into an upside-down trapezoid.

**Figure 2:**
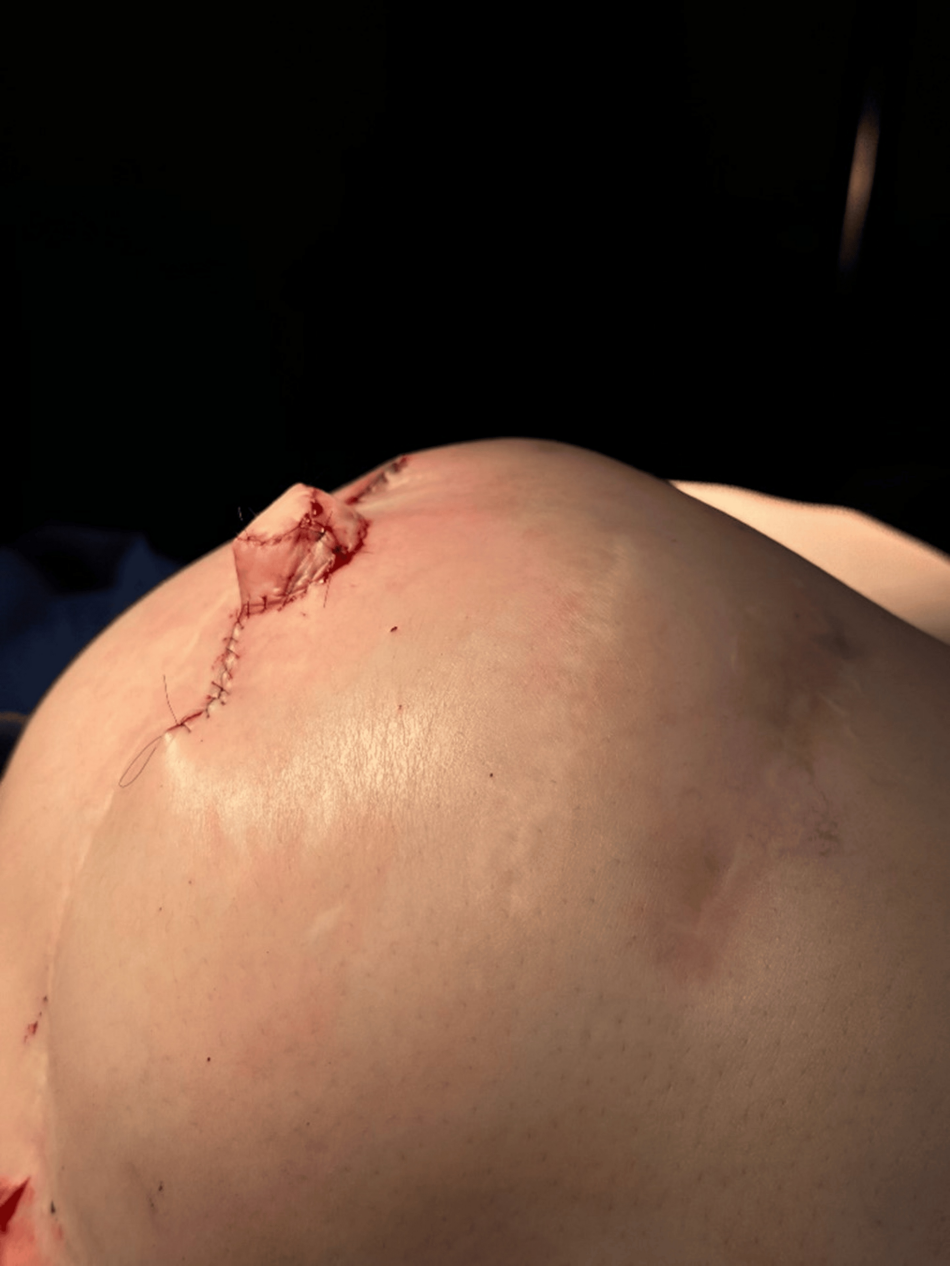
Lateral view of the C-V flap

**Figure 3:**
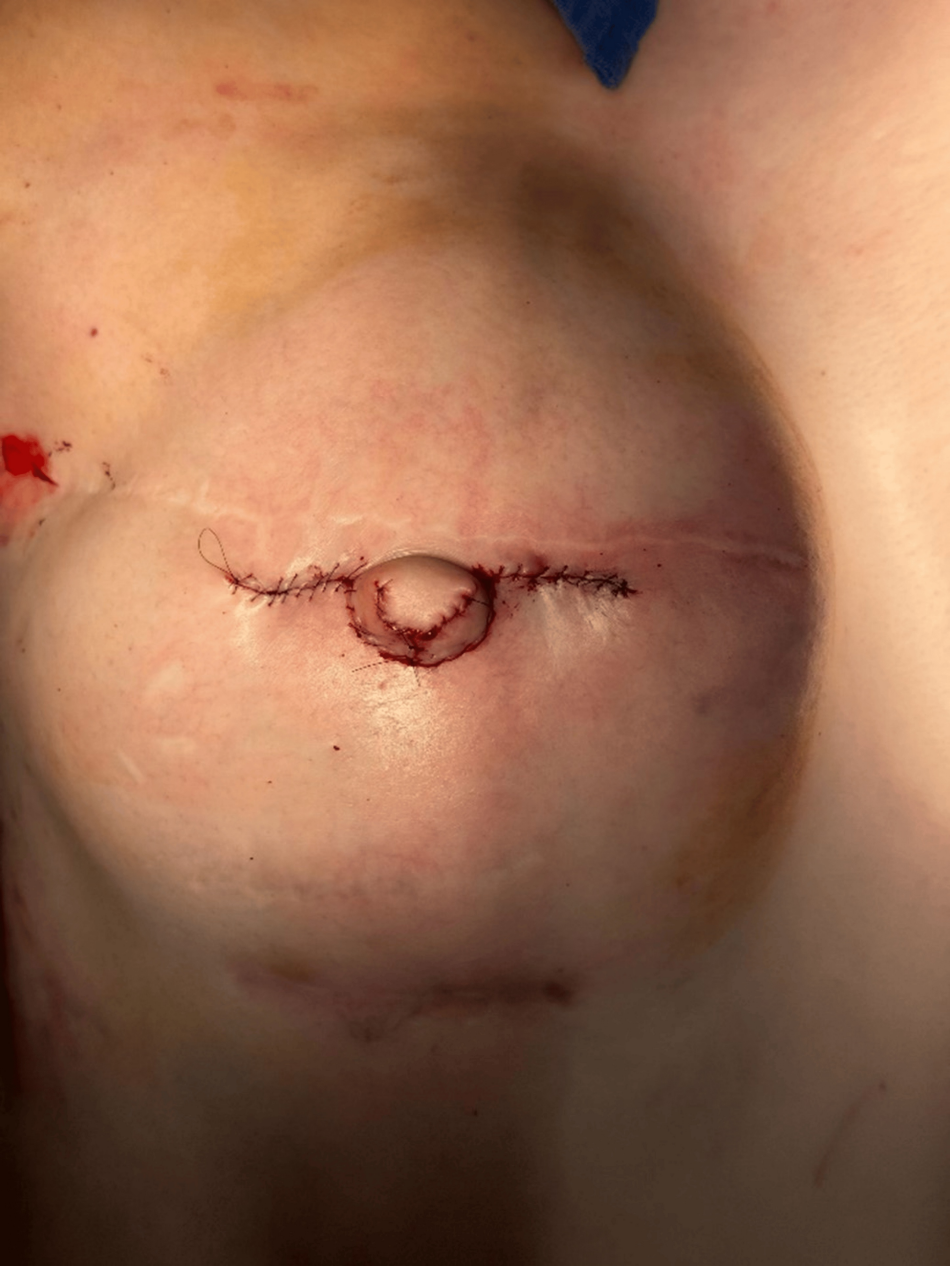
Frontal view of the C-V flap

To form the nipple column, the two V flaps were approximated and sutured at the base using 4-0 Monosyn. A 1.2 × 1.2 × 0.3 cm piece of acellular dermal matrix (ADM) (CGDerm, CGBio Corp., Seungnam, Korea) was inserted through the column and positioned at the base to act as a central pillar, supporting the projection. Finally, the C flap was approximated at the top and secured with 6-0 Prolene sutures, completing the reconstruction (Figure 2 and Figure 3).

## Discussion

Nipple reconstruction is a vital aspect of breast reconstruction that not only aims to restore the aesthetic appearance of the nipple-areola complex (NAC) but also addresses the psychological well-being of patients by providing a sense of completeness and body image restoration. Among the various methods for nipple reconstruction, local flaps such as the C-V flap have become widely recognized for their effectiveness in creating natural-looking results with relatively low complication rates. The C-V flap technique, in particular, is noted for its ability to preserve the aesthetic appearance of the reconstructed nipple by utilizing nearby tissue, which ensures better integration with the surrounding skin and tissue. However, one of the significant challenges associated with this technique is the gradual loss of nipple projection over time, often necessitating additional corrective surgeries to restore the desired outcome [1,2].

The loss of nipple projection after initial reconstruction is a common issue with many techniques, but the C-V flap's vulnerability to this problem has prompted various modifications to enhance its long-term effectiveness. The incorporation of purse-string sutures into the modified C-V flap technique represents a noteworthy innovation that aims to counteract this drawback. By placing sutures around the base of the nipple, the modified technique helps to stabilize the tissue and maintain projection for a longer period. This modification appears to significantly reduce the need for revision surgeries, addressing a critical concern for both patients and surgeons alike. Studies comparing the traditional C-V flap with the modified version indicate a marked improvement in the retention of nipple projection, with patients reporting sustained results at three, six, and 12 months post-surgery [7].

The impact of this modification on the overall quality of patient outcomes is substantial. For patients undergoing nipple reconstruction, the ability to retain nipple projection without the need for additional revisions not only improves the aesthetic result but also reduces the emotional and financial burden associated with multiple surgeries. Moreover, the improvement in projection retention is especially beneficial for patients who may be at higher risk of complications, such as those with prior radiotherapy or other treatments that compromise tissue viability. The modified C-V flap, with its stabilizing effect, provides a safer and more predictable outcome for these patients, further emphasizing its versatility and effectiveness as a technique for nipple reconstruction.

Furthermore, the use of composite grafts, such as those obtained from the contralateral nipple, can complement the C-V flap technique by enhancing both projection and symmetry. This approach is particularly advantageous when the contralateral nipple is large and has thin skin, allowing for better alignment with the reconstructed nipple. While grafting techniques like this can be associated with some risk of complications, such as graft failure, the combination of local flaps with grafts helps to minimize these issues by providing a more robust and natural result [3]. This holistic approach addresses the need for both functional and aesthetic restoration, ensuring that patients achieve a balanced and harmonious nipple-areola complex.

Additionally, tattooing remains a popular and effective method for areola reconstruction, offering a relatively low-risk procedure with satisfactory results in terms of aesthetics. Tattooing is often preferred over grafts because it carries a lower risk of complications, such as infection or graft failure, and is less invasive. However, for patients looking for a more complete restoration of the NAC, combining tattooing with techniques such as the C-V flap and composite grafts can provide a more comprehensive solution to the challenges of nipple-areola reconstruction [2,5].

## Conclusions

Nipple reconstruction is essential in breast reconstruction, restoring both aesthetics and psychological well-being. The C-V flap remains a reliable technique, with modifications such as purse-string sutures improving long-term projection and reducing revision rates. Enhancements such as composite grafts and tattooing further refine symmetry, accommodating individual anatomical variations and prior treatments. As techniques evolve, personalized approaches ensure optimal outcomes, minimizing complications and maximizing patient satisfaction.
